# Diagnostic utility of p63/P501S double sequential immunohistochemical staining in differentiating urothelial carcinoma from prostate carcinoma

**DOI:** 10.1186/1746-1596-6-67

**Published:** 2011-07-21

**Authors:** Malini Srinivasan, Anil V Parwani

**Affiliations:** 1 Department of Epidemiology, Graduate School of Public Health, University of Pittsburgh Cancer Institute, Pittsburgh, Pennsylvania, US; 2 Department of Pathology, University of Pittsburgh Medical Center, Pittsburgh, Pennsylvania, US

## Abstract

**Background:**

Distinguishing urothelial carcinoma (UC) from prostate carcinoma (PC) is important due to potential therapeutic and prognostic implications. However, this can be a diagnostic challenge when there is limited tissue and in poorly differentiated tumors. We evaluated the diagnostic utility of a dual immunohistochemical stain comprising p63 and P501S (prostein), applied sequentially on a single slide and visualized by double chromogen reaction, in differentiating these two cancers. Thus far, there have been no previous studies assessing the diagnostic utility of p63 and P501S combined together as a dual immunostain in distinguishing between these two cancers.

**Methods:**

p63/P501S dual-color sequential immunohistochemical staining was performed on archival material from 132 patients with high-grade UC and 23 patients with PC, and evaluated for p63 (brown nuclear) and P501S (red cytoplasmic) expression. Both the staining intensity and percentage of positive tumor cells were assessed.

**Results:**

p63 was positive in 119/132 of UC and negative in PC. P501S was positive in 22/23 of PC and negative in UC. The p63+/P501S- immunoprofile had 90% sensitivity and 100% specificity for UC. The p63-/P501S+ immunoprofile had 96% sensitivity and 100% specificity for PC.

**Conclusion:**

Our results indicate that double sequential immunohistochemical staining with p63 and P501S is highly specific and can be a useful tool in distinguishing UC from PC especially when there is limited diagnostic tissue as it can be performed on a single slide.

## Background

Distinction between prostate carcinoma (PC) and urothelial carcinoma (UC) is important due to the potential therapeutic and prognostic implications. Whereas hormone therapy may be used in treatment of PC, chemotherapy is used for UC. However, discriminating between these two cancers can be a diagnostic challenge especially in high grade tumors and in the presence of limited tissue. Immunohistochemistry, using both established and newer markers, is often used as a diagnostic tool in identifying the prostatic or urothelial origin of tumors.

Among the markers used to distinguish between urothelial and prostate cancers, prostate-specific antigen (PSA) and prostate-specific acid phosphatase (PSAP) are most commonly used to establish the prostatic origin of tumors; however, their expression is significantly decreased in poorly differentiated prostatic cancers [1,2]. Among the newer markers, prostate-specific membrane antigen (PSMA) and P501S (prostein) have been shown to have excellent specificity in differentiating prostate from urothelial cancers [3]. While alpha-methylacyl-CoA-racemase (AMACR), also known as P504S, is a useful biomarker of prostate cancer, it is also expressed in some non-prostate cancers including urothelial cancers [4] and therefore is not useful in making the distinction between PC and UC. Prostein is a prostate-specific 553 amino acid protein that was identified by cDNA subtraction in conjunction with high throughput microarray screening. It localizes to the cytoplasm, specifically to the Golgi complex, and its expression is restricted to prostatic tissue and unrelated to Gleason grade [5,6].

To establish urothelial differentiation, high molecular weight cytokeratin (HMWCK, clone 34βE12), thrombomodulin, cytokeratin (CK) 7 and CK 20 are commonly used in clinical practice. However, they often have to be used as part of an antibody panel and are not specific for UC [7,8]. Among other markers of urothelial origin are thrombomodulin, a transmembrane glycoprotein involved in intravascular coagulation, and uroplakin III, a transmembrane protein expressed in urothelial cells. While thrombomodulin is a sensitive urothelial marker, it is also expressed in a variety of other tumors [9]. Uroplakin III is highly specific, but only moderately sensitive in identifying UC [8,10]. More recently p63 has emerged as a marker of urothelial differentiation [7,11]. p63 is a transcription factor belonging to the p53 family that localizes to the nucleus and shares structural and sequence homology with p53, and has been shown in several studies to be a marker of urothelial origin of tumors [11-13].

The aim of this study was to evaluate the diagnostic utility of p63 and P501S in distinguishing between PC and UC using dual-color immunohistochemical staining performed on a single slide by sequentially applying the antibodies.

## Methods

### Cases

Our study was approved by the Institutional Review Board at the University of Pittsburgh. Archival material from 139 patients with high grade UC and 25 cases with PC from the Pathology Department at the University of Pittsburgh Medical Center (UPMC) was used in this study. The UC cases consisted of 132 high grade invasive UCs and 7 high grade noninvasive UCs from radical cystectomy, radical cystoprostatectomy, or transurethral bladder resections performed at UPMC between 1992 and 2008. Routine formalin-fixed paraffin-embedded (FFPE) whole sections were used in 23 UC cases, and the remaining 116 cases were distributed on two tissue microarrays (TMAs). The TMAs were constructed using a manual arrayer (Beecher Instruments Inc., San Prairie, WI). Two to 4 cores (core diameter 0.6 mm) were represented from each urothelial cancer case. Cores from adjacent normal appearing urinary bladder tissue and other anatomic sites were also included in the TMAs. However, only the cancer containing cores were scored for this study. For prostate cancer, we used routine FFPE whole sections from 25 patients who underwent radical prostatectomy for PC at UPMC between 2000 and 2007. None of the patients received neoadjuvant chemotherapy, radiation, or hormone therapy.

Of the 139 urothelial cancer patients, 6 cases on routine sections were excluded as there was no tumor and 1 case on TMA was excluded as there was no tumor present in both the cores obtained from this patient, after immunohistochemical staining. Among the 25 patients with prostate cancer, 2 cases were excluded as there was no tumor present on the slide after performing immunohistochemistry. The remaining 132 urothelial cancer cases and 23 prostate cancer cases [Gleason score 10 (n = 1), Gleason score 9 (n = 6), Gleason score 8 (n = 7), Gleason score 7 (n = 4), Gleason score 6 (n = 5) were included in the final analyses.

### Immunohistochemistry

Immunohistochemistry was performed on 5 micrometer sections of the FFPE routine sections and TMAs using monoclonal mouse antibodies against p63 (4A4, DAKO, Carpinteria, CA) and p501S (10E3, DAKO, Carpinteria, CA) applied sequentially. The sections were deparaffinized and hydrated, and heat induced epitope retrieval was performed using Borg decloaking high pH buffer in the Biocare decloaking chamber. Endogenous peroxidase activity was blocked with 3% hydrogen peroxide. The slides were first incubated with an avidin-biotin kit, followed by incubations with the p63 primary antibody (1:200 dilution) for 45 minutes, streptavidin-horseradish peroxidase, and Betazoid Diaminobenzidine for color development. Incubation with Denaturing solution for 5 minutes was done before application of the second primary antibody. P501S primary antibody (1:400 dilution) was then applied and incubated for 45 minutes, followed by incubations with alkaline phosphatase streptavidin, and Vulcan Fast Red chromogen. The slides were counterstained with Dako Hematoxylin, rinsed with water, dehydrated with alcohol and xylene, and coverslipped. Appropriate controls were included. All incubations were done at room temperature.

### Scoring and Evaluation

Immunohistochemical expression was assessed semi-quantitatively for staining intensity and percentage of positive tumor cells with brown nuclear staining (for p63) and red cytoplasmic staining (for P501S). Only moderate or strong staining in at least 5% of the tumor cells was considered positive. For the TMAs, a case was considered positive if at least one core showed positivity.

Immunohistochemical staining scores for p63 and P501S were individually compared between urothelial and prostate cancers, and a p-value less than 0.05 was considered statistically significant. The sensitivity, specificity, and predictive values for combined p63 and P501S immunostaining was also determined for both tumor types.

## Results

**Figure 1:**
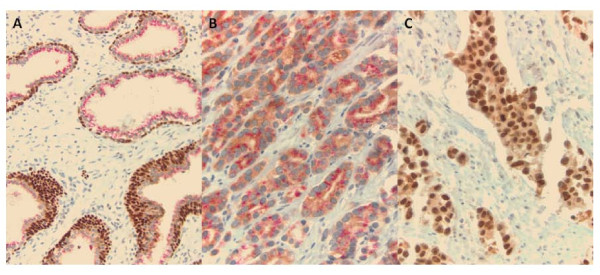
**Dual p63/P501S immunohistochemical stain**. **A**. Benign prostate glands with foci of basal cell hyperplasia showing red granular perinuclear cytoplasmic P501S staining and diffuse brown nuclear p63 staining of basal cell layer (X200.); **B**. Prostate cancer with red perinuclear cytoplasmic P501S staining and no brown nuclear p63 staining (X400); **C**. Urothelial carcinoma with diffuse brown nuclear p63 staining and absence of red P501S staining (X400).

The immunohistochemical findings are summarized in Table 1. Neither UC nor PC was positive for both p63 and p501S. Statistically significant p-values were observed for p63 and P501S expression, individually, in the distinction of UC from PC. One hundred nineteen of 132 (90.2%) UC were positive and none of the PC were positive for p63 (p < 0.0001). Twenty-two of 23 (95.7%) PC cases were positive and none of the UC cases were positive for p501S (p < 0.0001). p63 showed diffuse brown nuclear staining in urothelial cancers. p63 expression was also seen in the basal layer of the benign prostate glands included in some of the sections and this served as a positive internal control for p63 staining. P501S showed red granular perinuclear cytoplasmic staining of prostate cancer cells. Benign prostatic tissue included in some of the cases also showed P501S positivity (Figure 1).

**Table 1:** Immunohistochemical expression of p63 and P501S in urothelial carcinoma (UC) and prostate carcinoma (PC)

Immunohistochemical stain	UC, n = 132 (%)	PC, n = 23 (%)
p63	119/132 (90.2)	0/23 (0)

p501S	0/132 (0)	22/23 (95.7)

**Figure 2:**
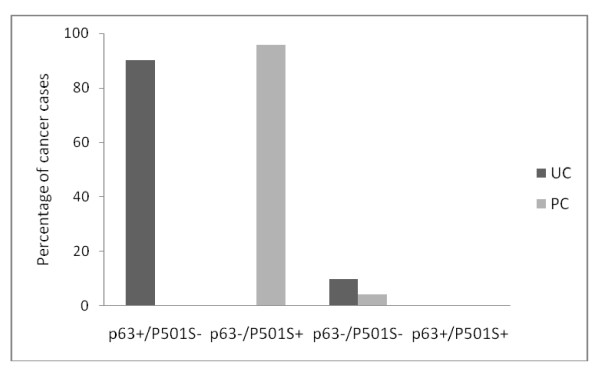
**p63/P501S combination immunohistochemical profile in urothelial carcinoma (UC) and prostate carcinoma (PC)**.

The p63 and P501S combination immunohistochemical profiles in UC and PC are illustrated in Figure 2. The sensitivity, specificity, and predictive values of p63 and P501S combination immunoprofiles for distinguishing PC from UC are shown in Table 2. The p63+/p501S- immunohistochemical profile was seen only in UCs (90.2% sensitivity, 100% specificity, and 100% positive predictive value) and the p63-/p501S+ imunohistochemical profile was seen only in PCs (95.7% sensitivity, 100% specificity, and 100% positive predictive value).

**Table 2:** Sensitivity, specificity, and predictive values: p63/p501S immunostaining in urothelial carcinoma (UC) and prostate carcinoma (PC)

Immunohistochemical profile	Sensitivity	Specificity	Positive predictive value	Negative predictive value
P63+/p501S- for UC	90.2%	100%	1	0.639
P63-/P501S+ for PC	95.7%	100%	1	0.992

## Discussion

Morphological distinction of UC from PC can often be a diagnostic challenge especially in poorly differentiated tumors. Additionally, serum PSA levels may be raised in urothelial cancers that infiltrate the prostate gland adding to the diagnostic dilemma. Immunohistochemistry is often used as a diagnostic tool to accurately distinguish between these two tumors and establish the final diagnosis [14]. The difficulty in making an accurate distinction is further compounded when there is only limited tissue available, such as in needle biopsies, cell blocks and fine needle aspirations with only small foci of carcinoma, when additional sections may have to be ordered and there may not be adequate tissue remaining to perform multiple immunohistochemical stains on separate slides.

Although the diagnostic utility of p63 and P501S in distinguishing between primary PC and UC have been individually evaluated previously [3,11,15], thus far, there have been no previous studies evaluating these two markers together either as a cocktail or applied sequentially. Our results indicate that dual-color immunohistochemistry with p63 and P501S applied sequentially shows excellent specificity for distinguishing UC from PC. The differential localization (diffuse nuclear for p63 versus granular cytoplasmic for P501S) combined with the double chromogen reaction facilitate easy visualization (brown for p63 and red for P501S) and enable quick and easy interpretation of the markers all in one slide. Additionally, this technique can be easily performed in the laboratory and conserves tissue as it is performed on a single slide. Thus, this immunostain could be a potentially valuable tool to aid in the distinction between these two cancers in the presence of limited tissue. However, in order to accurately characterize this double sequential immunostain, further studies comparing its performance with immunohistochemistry performed using the same antibodies individually as well as comparing its performance in prostate needle biopsies versus radical prostatectomies are required.

In the current study, p63 positivity was seen in 119/132 (90%) of UC cases. None of the PCs were positive for p63. Of the 13 UC cases that were negative for p63, 3 cases had micropapillary features. Our results are comparable to Kunju et al [15] who also found diffuse nuclear p63 positivity in 92% of their UC cases using the same p63 monoclonal antibody. Chuang et al [3] using the same p63 antibody found p63 in 83% of their UC cases. Similar to our study, Kunju et al and Chuang et al did not observe nuclear p63 positivity in any of their PCs. Thus, p63 appears to be a useful marker in distinguishing between UC and PC due to its high specificity for UC.

We found granular perinuclear cytoplasmic P501S expression in 22/23 (96%) PC cases. There was no difference in P501S staining intensity across PC cases according to Gleason scores. None of the UCs in our study showed P501S positivity. Our results are similar to Kalos et al [5] who found prostein expression in 111/118 (94%) primary and metastatic prostate cancers. They also found prostein to have excellent specificity with no expression detected in 4,635 normal and malignant non-prostatic tissues. Chuang et al observed P501S positivity in all of their 38 PC cases. These authors also found P501S to have high specificity with only 2/35 (6%) high grade UC showing focal weak positivity. Other studies [16,17] have also shown prostein expression to be a highly specific marker for identification of prostatic origin of tumors. The granular perinuclear cytoplasmic expression of prostein is an important feature in establishing the prostatic origin of tumors. A recent study by Lane et al [18] found moderate diffuse cytoplasmic P501S staining in 11% of urinary bladder adenocarcinomas.

The single P501S negative case in our study was diagnosed as a poorly differentiated prostatic adenocarcinoma (Gleason score 9). The focus of carcinoma seen on the immunostain slide of this case showed atrophic features and p63 negativity for basal cells. Although prostein expression was absent in this focus of carcinoma, adjacent areas of high-grade prostatic intraepithelial neoplasias (HGPIN) expressed prostein and also showed p63 reactivity in the basal cells. It has been previously described that the expression of AMACR, is absent or decreased in atrophic PCs [19]. It is possible that prostein expression may similarly be decreased in prostate cancer with atrophic features. This could lead to a potential diagnostic pitfall especially in rare cases of prostate cancer when p63 is aberrantly expressed in a non-basal distribution [20]. Due to the potential impact on clinical practice, further studies are required to validate our finding and determine if prostein immunoreactivity varies among specific morphological variants of PC.

Our findings indicate that p63/P501S dual immunostaining shows excellent specificity and good sensitivity in distinguishing urothelial from PC. A p63+/P501S- immunoprofile favors a diagnosis of UC with 90.2% sensitivity and 100% specificity, while a p63-/P501S+ profile establishes a diagnosis of PC with 95.7% sensitivity and 100% specificity. The p63-/P501S- profile was seen in 14 cases (9%), 13 of which were urothelial cancers. Caution should be exercised in interpreting a p63-/P501S- profile as lack of p63expression does not rule out UC. Kunju et al [15] using a panel comprising PSA, HMWCK, and p63 to distinguish between PC and UC in 26 diagnostically challenging cases found p63 positivity in 10/13 UCs. The 3 remaining p63 negative UCs were also negative for HMWCK, and they established the urothelial origin using CK 7 and CK 20 expression. In a study by Higgins et al [11] that included 321 bladder UCs (n = 238 high grade, n = 83 low grade) and 267 PCs, placental S100 (S100P) and GATA 3 emerged as markers associated with urothelial differentiation, and S100P was found to have higher sensitivity for UCs. They found p63 expression in 87% of UCs and only 0.4% of PCs. However, when the expression of S100P was also considered, 94.9% of all UCs expressed one or both markers, while none of their PCs expressed both p63 and S100P. Higgins et al concluded that the expression of S100P and p63 are partly complementary and when used in combination each marker may identify UC cases missed by the other. Some studies have shown high sensitivity for detection of UC with HMWCK (clone 34βE12) [21] and thrombomodulin [9]. Immunohistochemistry using a triple antibody combination of p63, P501S, and S100P or HMWCK or thrombomodulin may increase the sensitivity of the p63/P501S immunostain for detection of UC, and needs to be confirmed in future studies.

## Conclusion

In conclusion, our results indicate that double sequential immunostaining with p63/p501S is highly specific in distinguishing primary PC from UC, and support the potential clinical utility of this immunostain as a diagnostic tool in distinguishing between these two cancers in settings where only limited diagnostic material is available since it enables simultaneous evaluation of both markers on a single histologic slide. Using a triple immunostain by adding another sensitive urothelial marker may further improve the diagnostic performance of this immunostain.

## Competing interests

The authors declare that they have no competing interests.

## Authors' contributions

MS was involved in the design of the study, performed statistical and immunohistochemical analysis, and drafted the manuscript. AVP conceived the study, was involved in the design and immunohistochemical analysis, and edited the manuscript for intellectual content. All authors read and approved the final manuscript.

## Funding

This work was supported in part by the University of Pittsburgh Cancer Center Support Grant (CCSG) P30 CA047904 and Department of Pathology at the University of Pittsburgh Medical Center.
